# Ion channels in acinar cells in acute pancreatitis: crosstalk of calcium, iron, and copper signals

**DOI:** 10.3389/fimmu.2024.1444272

**Published:** 2024-11-13

**Authors:** Hanli Wang, Jianhua Gao, Lingling Wen, Kejun Huang, Huixian Liu, Linsheng Zeng, Zhongyi Zeng, Yuxiang Liu, Zhizhun Mo

**Affiliations:** ^1^ Emergency Department, Shenzhen Traditional Chinese Medicine Hospital, Shenzhen, Guangdong, China; ^2^ Key Laboratory of Quantitative Synthetic Biology, Shenzhen Institute of Synthetic Biology, Shenzhen Institutes of Advanced Technology, Chinese Academy of Sciences, Shenzhen, Guangdong, China; ^3^ University of Chinese Academy of Sciences, Beijing, China

**Keywords:** acute pancreatitis, ion channels, acinar cells, Ca^2+^, Fe^2+^, Cu^2+^

## Abstract

The initial stages of acute pancreatitis (AP) are characterized by a significant event - acinar ductal metaplasia (ADM). This process is a crucial feature of both acute and chronic pancreatitis, serving as the first step in the development of pancreatic cancer. Ion channels are integral transmembrane proteins that play a pivotal role in numerous biological processes by modulating ion flux. In many diseases, the expression and activity of ion channels are often dysregulated. Metal ions, including calcium ions (Ca^2+^), ferrous ions (Fe^2+^), and Copper ions (Cu^2+^), assume a distinctive role in cellular metabolism. These ions possess specific biological properties relevant to cellular function. However, the interactions among these ions exacerbate the imbalance within the intracellular environment, resulting in cellular damage and influencing the progression of AP. A more in-depth investigation into the mechanisms by which these ions interact with acinar cells is essential for elucidating AP’s pathogenesis and identifying novel therapeutic strategies. Currently, treatment for AP primarily focuses on pain relief, complications prevention, and prognosis improvement. There are limited specific treatments targeting acinous cell dedifferentiation or ion imbalance. This study aims to investigate potential therapeutic strategies by examining ion crosstalk within acinar cells in the context of acute pancreatitis.

## 1 Introduction

AP is an inflammatory condition that affects the pancreas and is a common gastrointestinal disorder, with an annual incidence of approximately 34 cases per 100,000 individuals (1). It is important to note that about 20-30% of people with AP will develop chronic pancreatitis (2). Acute and chronic pancreatitis is a common underlying disease in clinical practice (3). ADM is a prominent characteristic of both acute and chronic pancreatitis and may even serve as the initial trigger for the development of pancreatic cancer. Acinar cells undergo dedifferentiation and transition into a duct-like phenotype, referred to as ADM, as a protective response to stress in the pancreas. The mechanism enables acinar cells to survive even after losing their differentiation phenotype (3). This process occurs due to the high plasticity of acinar cells, which play a crucial role in pancreatic regeneration following mild damage (3, 4).

While the etiology of pancreatitis may be diverse, the immune response to cellular injury remains consistent. In the early stages of AP, aseptic inflammation of the pancreas leads to systemic inflammatory response syndrome (SIRS), which can vary widely in severity. Apart from providing supportive treatment until the inflammation subsides, no specific therapies are available to mitigate or prevent the condition (5). In recent years, as research on the pathogenesis of pancreatitis has advanced, potential intervention targets have been identified. For instance, the severity of SIRS is mitigated through the inhibition of inflammatory mediators released due to pancreatitis (6). Furthermore, specific immunomodulators have been identified to regulate the immune response and alleviate the systemic impact of pancreatitis (7).

Cellular ion signaling is crucial in numerous physiological processes, remarkably immune response to infections. An effective immune response relies on complex interactions of coordinated transcellular and intracellular signaling cascades. It identifies pathogen-associated molecular patterns (PAMPs) and injury-associated molecular patterns (DAMPs). It ultimately reaches its goal of establishing protective immunity and immune memory. In this process, various metal cations, such as Ca^2+^, Fe^2+^, and Cu^2+^, play a pivotal role in transmitting diverse signals (8). Ion signaling serves as a crucial mechanism for information transfer in acinar cells. Although these treatments remain in the research phase, they are potential future therapeutic options for AP.

## 2 Acinar cell remodeling in acute pancreatitis

Acinar cells are the primary cell type in the pancreas, accounting for 90% of all cells. Acinar cells typically secrete digestive enzymes such as amylase, trypsinogen, elastase, and carboxypeptidase A (9). Acinar cells have various inherent mechanisms to detect, mitigate, and regulate the microenvironment. This is essential for controlling the autolytic digestion of the numerous enzymes. These processes are critical to mitigating tissue damage and facilitating regeneration (10). The proenzymes, or zymogens, characteristic of the duodenum, are released to counteract the effects of the many enzymes secreted by acinar cells. Simultaneously, many enzymes are secreted along with trypsin inhibitors to prevent premature trypsinogen activation. Trypsinogen is only activated in the small intestine and activates other precursor digestive enzymes (11). Ultimately, acinar cells develop the capacity to undergo ADM.

**Figure 1:**
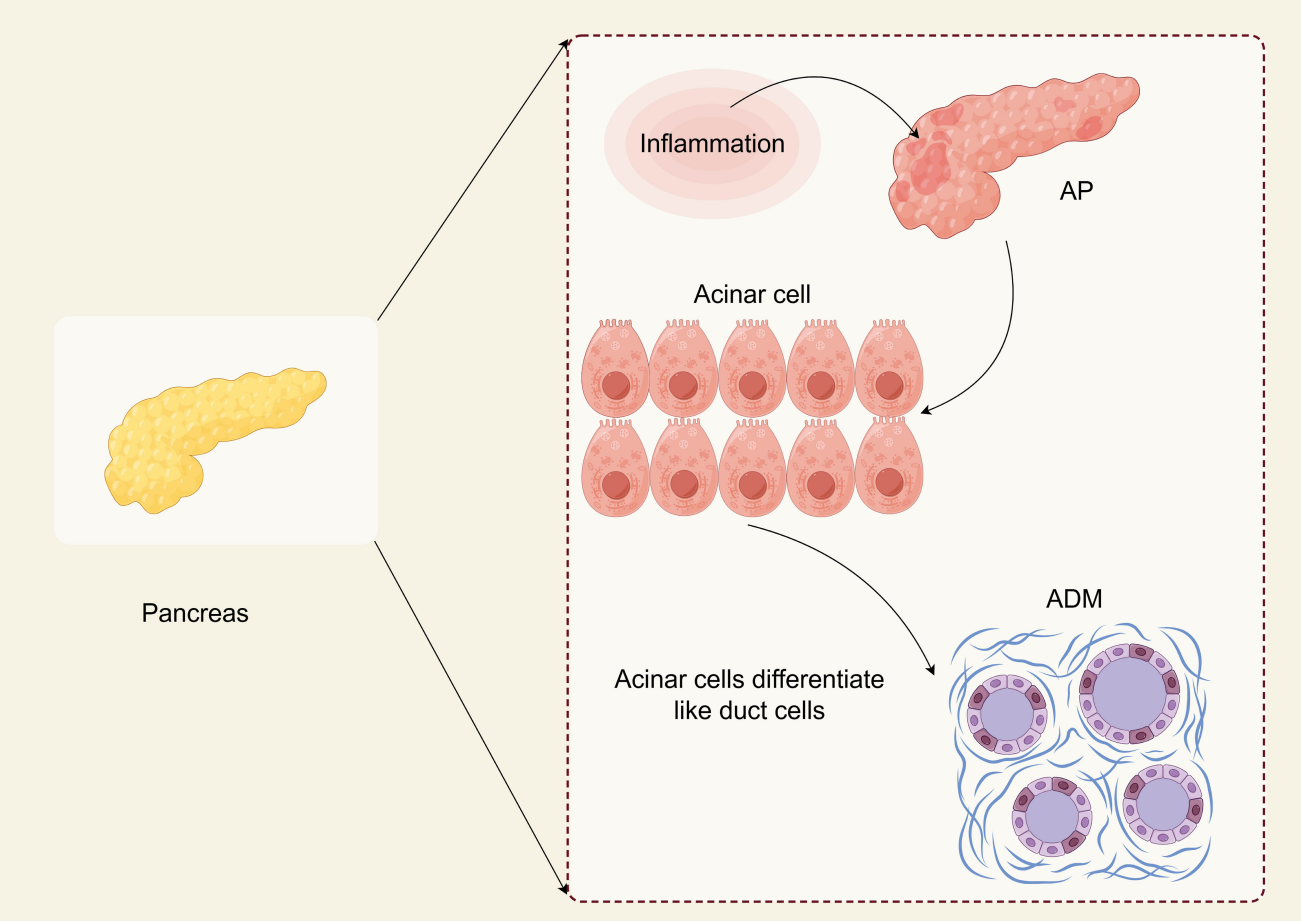
ADM in AP (By Figdraw). Acinar cells are the primary cell type of the pancreas. They exhibit high plasticity and can undergo a trans-differentiation process to form a progenitor cell-like cell type with ductal characteristics called acinar to ductal metaplasia (ADM). ADM is essential for pancreatic regeneration after injury and can be reversed once the damage has subsided. ADM may cause pancreatic intraepithelial neoplasia (PanIN), which is a typical precancerous lesion before pancreatic cancer. Understanding the intermediate state of ADM and the critical molecules that regulate ADM formation may help develop new prevention strategies that can not only target people with acute pancreatitis but also benefit those at high risk for pancreatic cancer.

ADM is a phenotypic transition from acinar cells to ductal cells that protect damaged cells or tissues from self-digestion (**Figure 1**) (12). During ADM, acinar cells undergo morphological changes and lose their original function, accompanied by alterations in gene expression related to cell differentiation, proliferation, and survival. This process is defined by the substitution of one cell type for another and is reversible. It can be triggered by various stimuli, including chronic inflammation or cellular damage (12, 13). This transformation leads to the adoption of duct-like cellular morphology and transcriptional alterations, mirroring the characteristics of embryonic progenitor cells (14, 15). It is crucial to note that ADM cannot simply be classified as a trans-differentiation event from acinar cells to ductal cells. This is because acinar cells undergo dedifferentiation into embryonic progenitor cell-like phenotypes before differentiating into ductal cells. The terminology surrounding metaplasia, transdifferentiation, and dedifferentiation remains controversial (16). ADM is believed to function as a protective mechanism that temporarily mitigates widespread tissue damage caused by excessive secretion of digestive enzymes. If the stimulating factor for metaplasia is removed, the damaged tissue may revert to its normal state. However, if the persistent stimuli promoting metaplasia exploit the plasticity of acinar cells, processes such as metaplasia, dedifferentiation, and transdifferentiation may lead to tumor development (17).

The hallmark pathological alteration in AP is the damage to pancreatic acinar cells, which results in the inappropriate activation of trypsinogen within these cells, ultimately initiating the autodigestion of pancreatic parenchyma (18). Research has shown that deceased and injured pancreatic acinar cells can release damage-associated molecular patterns (DAMPs), such as histones, DNA, and heat shock proteins. The accumulation of these pro-inflammatory DAMPs can subsequently activate the inflammatory response and release inflammatory factors. This process has the potential to worsen pancreatic damage and contribute to systemic inflammatory response syndrome (SIRS) and multi-organ failure (19). Concurrently, inflammatory cells are infiltrated, with macrophages and neutrophils being the first responders in the pancreas and contributing to tissue injury (20). The initial immune response is characterized by the secretion of pro-inflammatory cytokines, which can result in SIRS (21). Excessive inflammation at this time often leads to a condition associated with immunosuppression and potential secondary pancreatic necrosis called compensatory anti-inflammatory response syndrome (CARS) (22).

Despite the loss of some acinar cells due to necrosis and apoptosis during AP, others undergo ADM (17). Numerous observations provide compelling evidence for the existence of ADM. For example, experiments have shown that human pancreatic acinar cells can differentiate into ductal cells. Pancreatic ductal adenocarcinoma (PDAC) accounts for 95% of pancreatic tumors and primarily arises from pancreatic intraepithelial neoplasia (PanIN) (23). The formation of PanINs is attributed to the transdifferentiation of acinar cells (24). Introducing the Kirsten rat sarcoma viral oncogene homolog into murine acinar cells has induced significant ADM, progressing to PanIN and leading to PDAC (25). AP significantly contributes to chronic pancreatic disease; both conditions exhibit notable similarities while closely linked with the transformation of acinar cells into ADM. In summary, inhibiting the conversion process from acinar cells to ADM represents a crucial mechanism for managing the pathogenesis of acute pancreatitis and is also vital in preventing pancreatic cancer.

## 3 Calcium

The influx of Ca^2+^ is essential for advancing of the cell cycle and is pivotal in every phase of immune cell proliferation (26). In AP, the dysregulation of intracellular calcium ion signaling has emerged as a hallmark of the disease, leading to heightened generation of reactive oxygen species (ROS), impairment of mitochondrial function, activation of digestive enzymes within acinar cells, and cell death (27). As a result, preventing cellular Ca^2+^ overload and reducing its toxic effects has become one of the most promising therapeutic strategies (28).

### 3.1 The physiological function of Ca^2+^

Ca^2+^ signaling is pivotal in regulating pancreatic exocrine function, ensuring the appropriate secretion of digestive enzymes and fluids (29). The digestive enzyme prozyme is stored in apical granules of pancreatic acinar cells (PACs). It is synthesized through the polarization of the Golgi apparatus and condensing vacuoles (30). In this process, the endoplasmic reticulum and mitochondria act as critical regulators of Ca^2+^, responding to calcium signaling triggered by the release of endoplasmic reticulum storage in apical regions of cells. They enhance the production of adenosine triphosphate (ATP) required for secretion processes. Additionally, mitochondria buffer and regulate cytoplasmic calcium concentration by storing it in the matrix and controlling its slow release (29).

The Inositol 1,4,5-trisphosphate receptors (IP3R) function as an intracellular messenger that links the activation of G protein-coupled receptors with the release of Ca^2+^ (31). The findings indicate that the mobilization of intracellular Ca^2+^ is crucial for the exocytosis of digestive enzymes (32). This process is sensitive to changes in physiologically relevant concentrations, such as acetylcholine and cholecystokinin, produced in the endoplasmic reticulum at the apex of the cell. The connection between the apical Endoplasmic reticulum (ER) and the basal ER makes the latter an essential reservoir for repeated secretion of Ca^2+^ (33). Ca^2+^ peaks mediate the secretion bursts, and the wave-like changes are associated with the variations in IP3R receptor subtypes across different cellular regions. The apical region contains subtypes that exhibit sensitivity to low concentrations of IP3R and preferentially release Ca^2+^ (34). Simultaneously, the mitochondria of acinar cells, situated between the zymogenic granular region and the nucleus, respond to Ca^2+^ by activating complexes that enhance nicotinamide adenine dinucleotide (NADH) levels, drive ATP production, and support exocytosis as well as digestive enzyme secretion (35). Inhibition of ATP-dependent plasma membrane Ca^2+^ ATPase has been demonstrated to protect AP acinar cells by preventing cytotoxic Ca^2+^ overload (36).

### 3.2 The disturbance of Ca^2+^ metabolism in AP

Experimental observations of AP have demonstrated that increased Ca^2+^ concentration triggers a significant release of Ca^2+^ from the endoplasmic reticulum, resulting in a more pronounced and sustained elevation of Ca^2+^ levels (37–39). Importantly, deep discharge of endoplasmic reticulum (ER) storage leads to depletion of ER Ca^2+^, resulting in the activation of store-operated Ca^2+^ entry (SOCE) channels by the plasma membrane. This subsequently causes the release of Ca^2+^ into the cytoplasm through the termination of the IP3R signal (40, 41). In addition to pancreatic agonists, Ca^2+^ elevation can also be induced by bile salts and ethanol metabolites, leading to subsequent mitochondrial Ca^2+^ overload, acinar cell death, and other symptoms of pancreatitis (42–44). The calcium in the plasma membrane can activate the calcium-releasing protein 1 (Orai1), endoplasmic calcium sensor, and matrix interaction molecule 1 (STIM1). These proteins work together to regulate the influx of various cell types, including pancreatic acinar cells (45). Orai1 is a gene responsible for encoding the calcium release-activating calcium (CRAC) channel protein. When the levels of Ca^2+^ in the endoplasmic reticulum decrease, STIM1, located near the plasma membrane, undergoes polymerization and binds to Orai1 to open channels that allow Ca^2+^ to enter the cell (46). Piezo1, a calcium-ion intracellular pathway, has been discovered in pancreatic acinar cells (47). This channel is activated by prolonged mechanical stress on the cell, resulting in a sustained increase in Ca^2+^ levels, which leads to mitochondrial depolarization and, ultimately, cell death.

**Figure 2:**
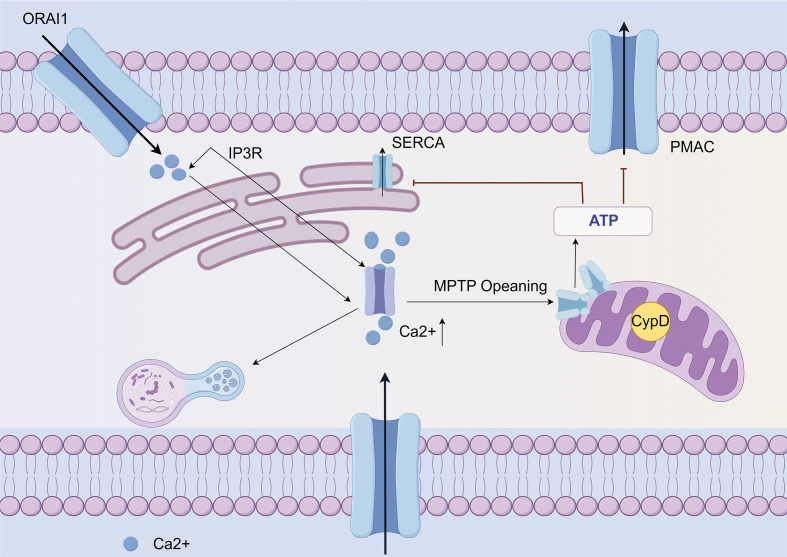
Ca^2+^ mediated mitochondrial dysfunction and cell death in AP (By Figdraw). Ca^2+^ release from the endoplasmic reticulum in acinar cells is mediated by the IP3R, triggered by multiple factors. Subsequently, the opening of Orai leads to an increase in overall calcium concentration. This elevation of calcium levels results in mitochondrial dysfunction and necrosis through the opening of the MPTP. The resulting ATP depletion compromises ATP-dependent mechanisms to reduce cytoplasmic calcium and exacerbates pathological calcium toxicity. Also, pathological calcium elevation activates other cytotoxic pathways, such as autophagy. Furthermore, activation of the PIEZO 1 mechanoreceptor promotes extracellular Ca^2+^ entry into acinar cells through its cationic channel properties.

Conversely, an excessive influx of Ca^2+^ through the Orai1 and Piezo1 Ca^2+^ channels results in mitochondrial dysfunction, ultimately leading to cellular apoptosis (48). Prolonged excessive release of Ca^2+^ leads to increased permeability of the mitochondrial inner membrane and results in cell death through both apoptosis and necrosis. The failure of mitochondria is caused by the sustained opening of the mitochondrial permeability transition pore (MPTP) induced by drugs (49). Inhibition of CypD through gene manipulation or drug intervention can effectively prevent the opening of the MPTP, which is the drug-induced mitochondrial permeability transition pore. This action plays a crucial role in preserving mitochondrial function and mitigating pancreatitis (50) (**Figure 2**). In the model of AP, it has been demonstrated that the inhibition of MPTP can maintain mitochondrial function.

## 4 Iron

The pancreas serves as the primary reservoir for iron storage, and there appears to be a significant correlation between iron-mediated cellular apoptosis and inflammation in the pathogenesis of AP. Injury to pancreatic tissue initiates an inflammatory cascade that increases ROS release and heightened lipid peroxidation. These mechanisms subsequently induce intracellular ferroptosis, which may further provoke additional inflammatory responses. This feedback loop can potentially exacerbate pancreatic tissue damage, thereby worsening the condition of AP.

### 4.1 The physiological function of iron

**Figure 3:**
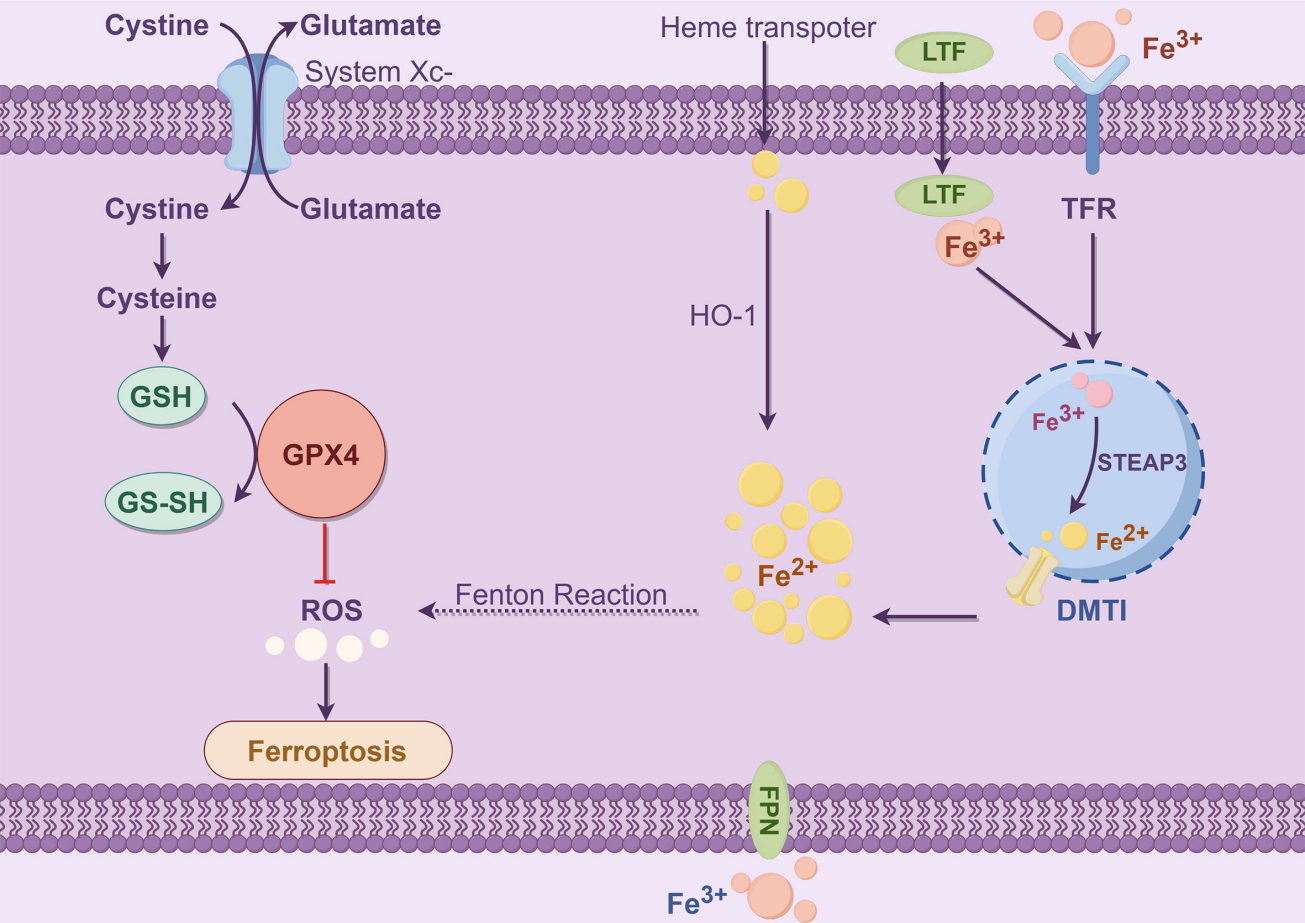
Iron metabolism (By Figdraw). The process of iron metabolism is that Fe^3+^ binds to TFR and then binds to TFR1 on the cell membrane to enter the cell. In the endosome, the iron oxidoreductase STEAP3 reduces Fe^3+^ to Fe^2+^, which is subsequently released by DMT1 into the LIP (ferritin stores stable iron, and the LIP stores unstable iron), creating a Fenton reaction that affects ROS levels and produces Ferroptosis. The Ferroptosis process depends on metabolite ROS and the transition metal iron. Intracellular and intercellular signaling events and environmental stresses can affect Ferroptosis by regulating cellular metabolism and ROS levels. The typical Ferroptosis control axis requires a cystine-glutamate antitransporter, specifying the system xc^−^, glutathione (GSH), which is a cofactor in the catalytic cycle of GPX4, which has a negative regulatory effect on Ferroptosis.

The majority of iron is obtained through food absorption and body circulation processes. Transferrin (TF) is a significant mode of iron transport, and TF is synthesized by the liver and released into serum globulins with strong Fe^3+^ binding capacity (51). In serum, Fe^3+^ binds to TF and is recognized by transferrin receptor 1 (TFR1) on the cell membrane, which promotes the transfer of transferrin into the endosome. Subsequently, in low-pH acidic endosomes, Fe^3+^ is released from the TFR1 complex and reduced to Fe^2+^ by six prostatic three transmembrane epithelial antigens (STEAP3) (52) (**Figure 3**). Finally, the bivalent metal ion transporter 1 (DMT1, SLC11A2) mediates the release of the endosome into the cytoplasm of the unstable iron pool (LIP) (53); thus, LIP and ferritin are intracellular storage forms. LIP induces oxidative stress-related toxicity and controls the cellular oxidative stress response element system by regulating the system balance (54). There is evidence that excess hinge accumulation in the LIP is one of the primary triggers of the Fenton reaction and can accelerate ferroptosis (55).

The study revealed elevated levels of pancreatic iron in mice induced by L-arginine, a conditionally essential amino acid, indicating that pancreatic iron overload may play a significant role in pancreatic damage (56). Moreover, an animal study revealed that a high-iron diet or the conditional knockout of glutathione Peroxidase 4 (GPX4) in the pancreas promoted experimental pancreatitis. In contrast, a ferroptosis inhibitor reversed this type of pancreatic inflammatory damage, suggesting a causative role for ferroptosis (57–59). Furthermore, increasing evidence indicates a reciprocal relationship between the exocrine pancreas and iron metabolism. The excessive iron accumulation during AP generates ROS, which induces dysfunction of pancreatic β cells and insulin resistance—ultimately leading to new-onset diabetes. In contrast, pancreatic β cells regulate iron levels through hepcidin secretion, and insulin controls iron absorption (60–62).

### 4.2 Ferroptosis in AP

The coping strategies of pancreatic acinar cells involve leveraging their plasticity and dedifferentiation capabilities. ROS is an inducer for the dedifferentiation of acinar cells (63, 64). In the initial stages of dedifferentiation, intracellular levels of ROS increase due to the upregulation of System xc^−^ cystine/glutamate antiporter (xCT, SLC7A11). However, as dedifferentiation progresses and xCT begins to decline, ROS levels remain elevated (65). The relationship between xCT and iron-induced cell death has been extensively researched in the context of cancer cells, including PDAC cells. The studies above have further elucidated the functional role of the cystine/glutamate reverse transporter system, xCT, which is typically upregulated in acinar cells and thus inhibits iron-induced cell death. While most research on pancreatic xCT has focused on suppressing tumor growth, this study offers insights into the pivotal role of xCT in non-cancerous tissue homeostasis and specifically in protecting pancreatic vesicle cells during stress, such as (experimental) pancreatitis.

The GSH/GPX4 pathway is known to suppress ferroptosis. It was demonstrated that treatment of cells with the ferroptosis inducer RSL-3 led to a significant increase in levels of inflammatory cytokines, including tumor necrosis factor (TNF) and interleukin-1 beta (IL-1β) (66). The expression and activity of GPX4 in cancer cells have been demonstrated to be regulated by TNF and IL-1β (67). Treatment of cells with TNF results in prolonged suppression of GPX4 and may also induce ferroptosis (68). It is well established that damaged acinar cells and activated immune cells in pancreatic tissue release reactive oxygen species, leading to an increase in malondialdehyde (MDA) and a decrease in superoxide dismutase (SOD) and GSH, all of which may further contribute to ferroptosis (69).

Without GSH, intracellular iron triggers lipid peroxidation, leading to cellular necrosis (70). Another study utilizing a mouse acinar cell line demonstrated that trypsin, a serine protease typically secreted by acinar cells, enhances cellular susceptibility to iron-induced damage. In animal models of azure-induced pancreatitis, mice with specific pancreatic GPX4 knockouts exhibited more severe symptoms (71).

## 5 Copper

Copper (Cu) is crucial in targeting and incorporating copper metalloenzymes (72). The uptake, transportation, and storage of copper in mammals are strictly regulated (73). Cuproptosis is a discovered form of regulated cell death triggered by excess Cu^2+^. Recent research has challenged the traditional understanding of copper-related signaling pathways, transcription factors, and biological processes (74). Therefore, removing copper could become a new strategy (75–77).

### 5.1 Cu metabolism and cuproptosis

**Figure 4:**
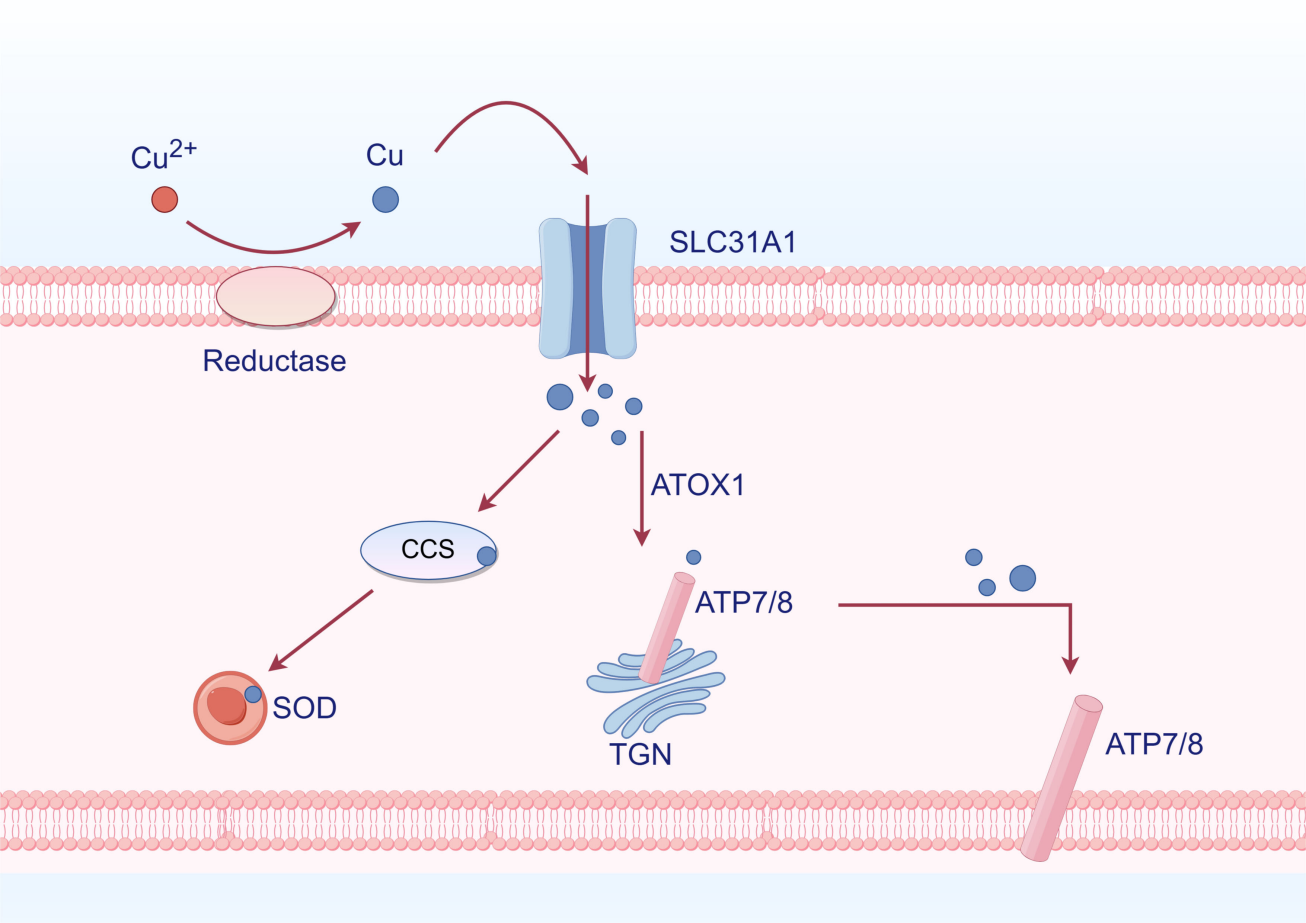
Cu metabolism (By Figdraw). Cu^2+^ is reduced to Cu by reductase outside the cell, subsequently entering the cell through SLC31A1. Inside the cell, it binds to Cu chaperones CCS and SOD, allowing for its distribution to specific cellular compartments such as the trans-Golgi network (TGN) and mitochondria. ATOX1 functions as an antioxidant that facilitates Cu transport to ATPases. Within the TGN, Cu-transporting ATPases ATP7A and ATP7B mediate the transfer of Cu from the cytoplasm into the TGN lumen, activating Cu-dependent enzymes in the secretory pathway. When intracellular levels of Cu are elevated, ATP7A and ATP7B exit from the TGN to promote Cu efflux.

Cu is widely recognized as a static cofactor, necessitating burial and protection within the enzyme’s active site. This traditional function of Cu exploits the redox potential of this transition metal to enhance enzyme activity in various processes such as energy maintenance, metabolism, and compound synthesis (78). Additionally, superoxide dismutase (SOD1) and its cofactor Cu chaperone for superoxide dismutase (CCS) contribute to cellular defense against oxidative stress to a certain extent. Cu also plays a role in the enzymatic synthesis of biological compounds (79). For example, ATOX1 is an antioxidant that transports Cu to ATPase. In the secretory pathway, the copper transport protein alpha or beta (ATP7A/B) binds copper to the newly folded protein (78). In the investigation of Cu, it was discovered that Cu functions as a dynamic signaling molecule. The protein kinase regulated by Cu was initially identified as MEK1/2, followed by other kinases such as ULK1/2. These two kinases are commonly upregulated in cancer cells. Cu’s regulation of these kinases suggests the potential use of chelation therapy to target MEK1/2 and inhibit MAPK activity, as well as ULK1/2 to inhibit autophagy signaling (**Figure 4**) (80).

Furthermore, it has been discovered that Cu plays a crucial role in inhibiting the dynamic action of phosphodiesterase (PDE) in degrading cyclic AMP. This discovery is essential for regulating the CAMP-dependent lipolysis process (81). Recent research has demonstrated that the H3-H4 proteins can catalyze the reduction of Cu^2+^ to Cu during their interaction, which holds significant implications (82). The REDOX activity of H3-H4 tetramers exerts a crucial influence on numerous Cu-dependent processes, including SOD1 function, and is thus indispensable for maintaining intracellular Cu homeostasis (82). While further characterization of the diverse roles of Cu in biology is still required, we have gained some insight into its involvement in dynamic processes (74).

Interestingly, Cu levels are increased in inflamed and malignant tissues (83). Inflammatory cytokines such as IL-17 promote cellular uptake of Cu by inducing the metal STEAP4. Following Cu absorption, E3 ligase XIAP is activated to enhance IL-17-mediated NF-κB activation while inhibiting caspase three activity. This also establishes a solid foundation for further research on Cu in AP (83).

In 1978, Chan demonstrated that elevated Cu concentrations result in cellular apoptosis (84). Disruption of Cu^2+^ homeostasis triggers cytotoxicity and induces cell death through various pathways, including accumulation of ROS, proteasome inhibition, and mitochondrial dysfunction. During mitochondrial respiration, Cu^2+^ binds to fatty acylated proteins in the tricarboxylic acid cycle, leading to fatty acylation modifications (85). The aggregation of iron-sulfur tuft in protein results in the downregulation of iron-sulfur tuft in expression, leading to the induction of protein-toxic stress and ultimately culminating in cell death (85). However, further exploration of the Cu-induced cell death phenotype and its signaling cascade’s regulatory mechanism is required.

There is no direct evidence linking Cu or cuproptosis to ADM in AP. However, the involvement of iron metabolism is well-established, and interactions between iron and copper have been documented. This discussion will elaborate on the theoretical implications of the interplay between copper and iron in AP while speculating on the role of copper metabolism to pave the way for future research.

## 6 Ion channel crosstalk

### 6.1 Crosstalk of calcium metabolism and iron metabolism

**Figure 5:**
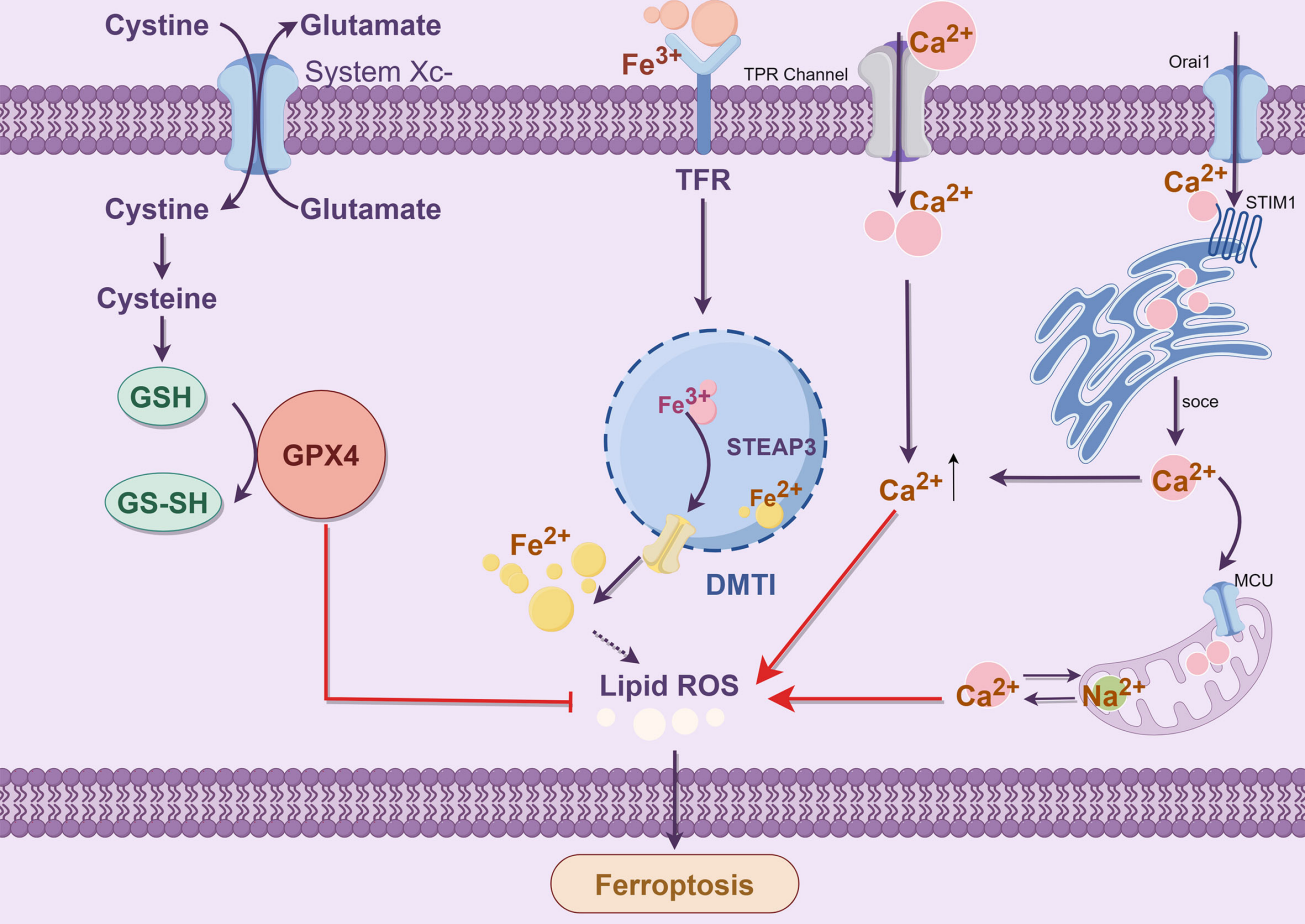
Crosstalk between Ca^2+^ and Fe^2+^ (By Figdraw). The endoplasmic reticulum calcium receptor STIM1 interacts with Orai1 to facilitate the SOCE mechanism for enhanced calcium influx. The transporter microcontrollers facilitate the transfer of cytoplasmic calcium into and out of mitochondria through the mitochondrial sodium-calcium exchanger Na^+^/Ca^2+^ Exchangers (NCX). Extracellular Ca^2+^ influx triggers the opening of permeable pores in the mitochondrial double membrane, leading to mitochondrial swelling and rupture. Ultimately, releasing Ca^2+^ from the mitochondrial intermembrane pool can overwhelm cytoplasmic levels and lead to cell death due to reactive oxygen species accumulation.

Disruption of calcium homeostasis can lead to cell death, such as ferroptosis. The influx of extracellular Ca^2+^ is primarily attributed to the depletion of intracellular glutathione, the generation of reactive oxygen species within the cytoplasm, and mitochondrial dysfunction (86). In a study, Ca^2+^-mediated cell death was inhibited by the cystine/glutamate reverse transport system xc^-^ (87). This oxidative glutamate or oxygen poisoning is known as ferroptosis (88). Iron chelating agents can effectively inhibit iron-mediated death and glutamate oxidation toxicity. Ferrostatin 1 can inhibit glutamate-induced oxidative toxicity (89). Maher discovered that specific compounds could mitigate oxidative glutamate toxicity by inhibiting mitochondrial ROS production or attenuating Ca^2+^ influx, thereby protecting against cell death induced by erastin or sulfamyridine (88). There was a time-dependent decrease in glutathione levels after the extracellular addition of glutamate to inhibit system xc- (90). The levels of ROS increase exponentially, leading to a surge in the activation of the signaling pathway that results in a CGMP-dependent influx of Ca^2+^ and subsequent cell death (91). The consensus is that the influx of Ca^2+^ plays a pivotal role in cellular apoptosis. The mechanism of calcium-induced oxidative toxicity of glutamate was investigated. Calcium sensitivity in glutamate-resistant was analyzed, and the difference was attributed to the downregulation of the Ca^2+^ channel Orai1 rather than the Ca^2+^ sensor STIM1 or STIM2. This regulation led to a significant reduction in Ca^2+^ entry into SOCE for storage operations (92) (**Figure 5**).

### 6.2 The crosstalk between iron metabolism and copper metabolism

**Figure 6:**
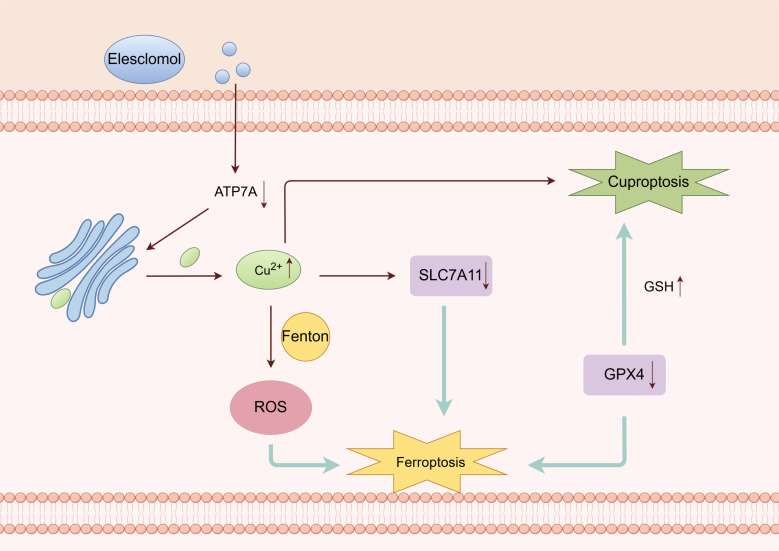
Crosstalk between ferroptosis and cuproptosis (By Figdraw). Cu chelating agent can induce the degradation of ATP7A, which promotes the efflux of Cu^2+^. Excessive Cu^2+^ can cause the Fenton reaction, ROS accumulation, then ferroptosis. Deletion of ATP7A leads to downregulation of SLC7A11. GPX4 cannot effectively inhibit oxidative stress and may further induce ferroptosis. The reduction of GSH due to GPX4 may further induce cuproptosis.

One study found that Cu^2+^ also triggers ferroptosis (93). Elesclomol-induced Cu chelation enhances ferroptosis (94) (**Figure 6**). The Cu chelating agent Elesclomol alone induces the degradation of ATP7A, which is responsible for facilitating Cu efflux (94). Excessive copper retention can cause the Fenton reaction, generating ROS and triggering ferroptosis (95). Conversely, ATP7A plays a protective role in preventing the degradation of SLC7A11. The loss of ATP7A mediated by Elesclomol results in the downregulation of SLC7A11 and an insufficient supply of cystine in cells. As a result, GPX4 is unable to inhibit oxidative stress effectively and may further induce ferroptosis (94).

Furthermore, Qian discovered that Cu^2+^ directly interacts with the cysteine residues C107 and C148 of the GPX4 protein, leading to the aggregation of GPX4. These aggregates are then recognized by the autophagy receptor and degraded through the autophagy pathway. Subsequently, autophagy induces ferroptosis (94).

## 7 Therapeutic strategies of calcium, iron and copper metabolism in AP

Currently, the predominant therapeutic strategy for AP primarily focuses on symptomatic management. Immediate intravenous fluid resuscitation is critical for AP patients to prevent organ dysfunction in cases of mild to moderate AP (96). Some studies suggest that lactated Ringer’s solution may offer advantages over normal saline in fluid therapy, but more extensive validation through larger clinical trials is required (97). Analgesic treatment, particularly with opioids, is also recommended for managing pain in AP patients (98). Nutritional support plays a crucial role in treating AP, with early initiation of enteral nutrition being preferred if tolerated by the patient, supplemented by parenteral nutrition when necessary to meet caloric demands (99). Close monitoring and timely intervention targeting pulmonary, cardiac, and renal involvement are vital during clinical diagnosis and treatment of AP (100). Antibiotic therapy may not be requisite as it does not diminish the risk of infectious pancreatic necrosis; empirical coverage should encompass gram-negative and anaerobic bacteria when deemed appropriate (101). Traditional Chinese medicine has been effectively employed in Asia for several decades as an adjunctive treatment for acute pancreatitis, with numerous studies substantiating its efficacy in alleviating symptoms, managing complications, and reducing mortality rates (102–104). According to bibliometric analysis, Dachengqi Decoction emerges as the most extensively researched TCM formulation within this domain—a promising avenue for future research (105).

The ongoing investigation into ion crosstalk in AP provides a novel perspective on its therapeutic approach. The inhibition of altered Ca^2+^ kinetics presents significant therapeutic potential for AP management. Preclinical studies utilizing experimental models have demonstrated that the blockade of Orai1 function, which preserves normal Ca^2+^ dynamics in acinar cells, effectively prevents mitochondrial dysfunction and the onset of pancreatitis (106). The specific agent CM4620, also referred to as Auxora, is currently undergoing clinical trials, underscoring its considerable significance (45). As a subsequent step, the company is actively conducting a Phase II trial—an open-label dose-response study—to evaluate the safety and efficacy of CM4620-IE in patients with acute pancreatitis accompanied by SIRS (107).

The activation of TRPM2 enhances the influx of extracellular Ca^2+^ in acinar cells triggered by bile acid, resulting in necrosis both *in vitro* and *in vivo*. In a biliary acute pancreatitis model, the knockout of the TRPM2 gene mitigated disease severity and conferred protection to acinar cells (108). Fourgeaud identified that JNJ-28583113, a potent inhibitor of TRPM2, offers cellular protection against oxidative stress and pro-inflammatory stimuli (109). However, its application *in vivo* is limited by drug metabolism. Additionally, TRPM2 represents a promising drug target for central nervous system disorders, with expectations for the identification of further inhibitors.

Targeting iron pathways may offer new opportunities for treating acute pancreatitis, such as Artesunate (110, 111) and Gemcitabine (112), which exert their effects on PDAC through the induction of ferroptosis induction. A significant challenge remains in developing pharmacological agents capable of modulating the iron sag pathway—either as monotherapies or in combination regimens—while minimizing adverse side effects and ensuring precise targeting. Under treatment selection pressure, resistance to therapy may develop, underscoring the necessity to elucidate the relationship between molecular characteristics and specific drug responses. Although clinical trials investigating iron death-dependent therapeutic strategies are currently lacking, preclinical studies remain indispensable.

Several strategies have been developed to modulate intracellular Cu and iron levels based on the significance of these metals in disease. One of the primary objectives of these interventions is to innovate new anti-cancer therapies, as iron and Cu play crucial roles in tumorigenesis and cancer progression. Specifically, cancer cells exhibit a heightened demand for iron compared to normal cells, often described as “iron addiction” (113). The treatment can be achieved through two diametrically opposite approaches: one involves the deprivation of iron and Cu from cells, while the other intentionally induces an excess of iron and Cu in cancer cells, leading to the generation of ROS and subsequent induction of cell death (114). Both mechanisms specifically target cancer cells, suggesting that consumption and supplementation of iron/Cu may be viable treatments. Furthermore, these treatment strategies also apply to a wide range of diseases.

Targeting strategies for iron and Cu have been extensively utilized, such as the widespread use of classic iron chelators for treating iron overload disorder, including delarolex, deferiprone, deferrable, deferoxamine, and tropine (115). The toxicity levels and therapeutic efficacy of these substances vary; however they are limited in their therapeutic effects (116). A potential countermeasure could involve the utilization of iron- and Cu-binding proteins in conjunction with chemotherapy drugs to enhance the specificity of drug delivery (117). Nanomedicines are an emerging and precise anticancer strategy for regulating iron and Cu levels. Furthermore, combining these methods may offer a more practical approach to treating patients and mitigating the toxic effects of iron and Cu. For instance, the utilization of curcumin in facilitating intracellular copper transport has been identified (118). Nanotechnology enhances the efficiency of curcumin nanoparticles by increasing their water solubility and specificity (119).

## 8 Conclusion

AP is associated with high morbidity and mortality in clinical practice. Currently, the most effective interventions for AP include symptomatic treatment and nutritional support. However, recent studies have identified calcium as a crucial factor in the pathogenesis of AP, making it a potential therapeutic target. Calcium metabolism also plays a significant role in AP and is closely linked to iron and copper metabolism. Acinar dedifferentiation is a critical pathological process in the progression of AP, and effective intervention can help delay its advancement. Interventions targeting ion-related signaling pathways are considered essential for preventing acinar differentiation. Nevertheless, gaps in our understanding of iron and copper metabolism still require further investigation. By addressing these scientific questions, we aim to gain a more comprehensive understanding of AP’s physiological and pathological mechanisms, thereby creating new translational opportunities for optimizing prevention, diagnosis, and treatment strategies that will ultimately improve the survival and quality of life for patients with AP.
